# Immune Thrombocytopenia in a Challenging Case of Disseminated Tuberculosis: A Case Report and Review of the Literature 

**DOI:** 10.1155/2010/946278

**Published:** 2010-09-26

**Authors:** Ankur Kalra, Ankit Kalra, Chandrasekar Palaniswamy, Naval Vikram, G. C. Khilnani, Rita Sood

**Affiliations:** ^1^Department of Medicine, All India Institute of Medical Sciences (AIIMS), New Delhi 110029, India; ^2^Department of Internal Medicine, Cooper University Hospital, Camden, NJ 08103, USA; ^3^Department of Medicine, Kalra Hospital & SRCNC, New Delhi 110015, India

## Abstract

Mycobacterium tuberculosis (MTB) continues to predominate the cause of morbidity, and mortality in the developing world. The disease affects all the organ systems, and presents in various pathologic disease states. We report an uncommon manifestation of this rather common infectious disease in a 19-year-old male. Immune-mediated thrombocytopenia occurring as a consequence of the tuberculosis infection itself is an exceedingly rare occurrence, and at the time of writing of this paper, only 15 such published reports exist in the English literature so far.

## 1. Introduction

Mycobacterium tuberculosis (MTB) continues to predominate the cause of morbidity and mortality in the developing world. The disease affects all the organ systems and presents in various pathologic disease states. We report an uncommon manifestation of this rather common infectious disease in a 19-year-old male. This case was challenging not only due to a rare manifestation of MTB, but also because the patient could not tolerate the classic first-line four-drug antituberculous therapy (ATT) and was managed with a five-drug modified ATT.

## 2. Case Report

A 19-year-old male with no known comorbidities presented to outside hospital with a history of high-grade fever of two weeks duration, associated with productive cough with scant expectoration. The clinical examination was significant for decreased air entry on right side of the chest on auscultation, with a stony dull percussion note. The patient had an unremarkable complete blood count and basic metabolic panel. Erythrocyte sedimentation rate was raised at 40 mm/hour. A chest x-ray done revealed blunting of the right costo-phrenic angle consistent with right-fsided pleural effusion. A diagnostic thoracentesis was performed that revealed an exudative, lymphocytic-predominant pleural fluid. A clinical diagnosis of tuberculosis involving the pleura was made, and patient initiated on four-drug ATT with isoniazid (INH), rifampin, pyrazinamide (PZA), and ethambutol (ETH). The ATT was modified several times, as the patient had significant side effects to many drugs including PZA causing high uric acid levels, ofloxacin causing severe gastrointestinal symptoms, streptomycin causing otologic toxicity manifesting as vertiginous symptoms, and ethambutol causing bilateral optic neuritis manifesting as bilateral diminution of vision, objectively confirmed by visual evoked potentials. While on modified ATT, the patient had a generalized tonic-clonic seizure. Magnetic resonance imaging (MRI) of the brain done revealed right frontal, temporal, parietal, and cerebellar parenchymal lesions. Antiseizure therapy with phenytoin was initiated. A follow-up chest x-ray following two months of ATT now revealed bilateral pleural effusions. A repeat diagnostic thoracentesis was consistent with an exudative, lymphocytic-predominant pleural fluid, with a high adenosine deaminase (ADA) content of 135.2 (normal <40). Bronchoscopy with bronchoalveolar lavage smear was negative for acid fast bacilli. A transbronchial lung biopsy showed multiple ill-defined epithelioid granulomas with inflammatory cell infiltrates.

The patient was referred to us with concerns for persistent moderate- to high-grade fever, cough with scant expectoration, worsening bilateral pleural effusions, and new onset epistaxis. Physical examination was significant for a febrile, tachycardic cachectic young male with florid petechial rash present over the chest and the anterior abdominal wall. There was a decreased expansion of both halves of the chest with decreased air entry bilaterally on auscultation. Examination of the abdomen was remarkable for hepatosplenomegaly. Review of other systems was unremarkable. Laboratory evaluation done revealed a low hemoglobin of 7.6 g/dL, a low platelet count of 29000/cu.mm, and deranged liver function tests with mild elevation of aspartate (AST), and alanine aminotransferase (ALT) (87, and 68 units/L), respectively and significantly high alkaline phosphatase (ALP) levels (1743 U/L). Patient had normal prothrombin time (PT) and activated partial thromboplastin time (aPTT). Other laboratory investigations done to rule out other causes of persistent fever despite adequate antituberculous therapy were all unremarkable, that is, a normal urinalysis, negative blood, and urine cultures, no malaria parasite on peripheral smear, negative dengue serologies, a negative Widal agglutination test, negative antistreptolysin O (ASO) titers, and a normal 2-dimensional echocardiogram. A contrast-enhanced computed tomography (CECT) scan of the chest and abdomen done revealed multiple miliary nodules seen throughout the lung parenchyma bilaterally, with bilateral pleural effusions, enlarged necrotic lymph nodes in subcarinal, and right hilar regions, multiple well-defined hypodense lesions noted in the liver, spleen, and right kidney, and large wedge-shaped hypodense lesion in the posterior surface of lower pole of spleen. A diagnosis of disseminated tuberculosis was made on clinical and radiologic evidence, and previous histopathologic data, and patient continued on modified ATT with the following five-drug regimen: INH, PZA, ethionamide, moxifloxacin, and amikacin. Other supportive measures were also instituted, including corticosteroids for disseminated tuberculosis (hydrocortisone sodium succinate 50 mg intravenously thrice daily) and platelet transfusions for a low platelet count.

**Figure 1:**
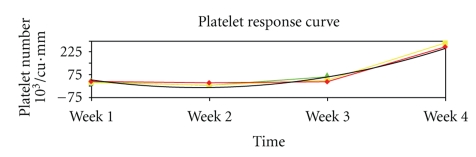


The patient's hospital course was complicated by a persistently declining platelet count, refractory to platelet transfusions. The peripheral smear examination revealed thrombocytopenia. Bone marrow aspirate showed a slight increase in megakaryocytes, consistent with megakaryocytic thrombocytopenia. Bone biopsy revealed multiple necrotizing epithelioid cell granulomas with areas of diffuse necrosis consistent with disseminated tuberculosis. The patient was initiated on 1 mg/kg/day of oral prednisone. The platelet count showed rapid recovery following initiation of immunomodulatory therapy, with a platelet count of 285000/cu.mm after a week of prednisone (Figure 1). A close followup was maintained, and the patient successfully weaned from prednisone with complete recovery of platelet count.

## 3. Discussion

Tuberculosis is one of the world's most challenging communicable diseases. The hematological abnormalities associated with tuberculosis are well recognized. Table 1 summarizes the hematological manifestations in patients with tuberculosis in various studies [1].

Bone marrow changes have also been well described in tuberculosis and include changes in marrow cellularity manifesting as myeloid hyperplasia that occurs more commonly with pulmonary tuberculosis, plasmacytosis, megaloblastic changes in cell lineages, and marrow aplasia. Miliary tuberculosis is associated with histiophagocytosis of all cell lineages and caseating granuloma formation in 60–70% cases. Other marrow changes in tuberculosis include marrow necrosis and myelofibrosis.

Thrombocytopenia in tuberculosis can occur due to a defect in platelet production (marrow suppression), as a side effect of anti-tuberculous therapy, histiophagocytosis as described above, tuberculosis complicated by thrombotic thrombocytopenic purpura (TTP), or disseminated intravascular coagulopathy (DIC), or due to immune-mediated platelet destruction. 

Drug-induced thrombocytopenia (i.e., as a side effect of anti-tuberculous therapy) develops within 6-7 days in individuals taking drugs for the first time, and within hours in sensitized patients. Some patients may not develop thrombocytopenia form months to years. The platelet count is usually restored within a week of cessation of the offending agent. Corticosteroids have also been used in such scenarios, with intravenous immune globulin (IVIg) or plasmapheresis reserved for life-threatening situations.

Immune-mediated thrombocytopenia occurring as a consequence of the tuberculosis infection itself is an exceedingly rare occurrence, and at the time of writing of this paper, only 15 such published reports exist in the English literature so far [2–16]. The immune basis of tuberculosis-induced thrombocytopenia in these case reports is supported by the presence of either the platelet antigen-specific antibodies or platelet surface membrane IgG [8, 10], or by response to immunomodulatory therapy [3, 6]. The pathogenesis of tuberculosis causing immune thrombocytopenia is thought to be the generation of antiplatelet antibodies by lymphocytes borne as a result of clonal proliferation due to host's immune response to the tuberculous pathogen [10]. In our patient, this was proven by favorable response to immunomodulatory therapy with concomitant ATT. Antiplatelet antibodies were not done as this was not routinely available for clinical use in India.

All the patients cited in the literature responded to immunomodulatory therapy with concomitant ATT. The facts that immunomodulatory therapy alone in all the reported cases did not result in improvement and that effective concomitant ATT was needed as an adjunct support tuberculosis as the cause for immune thrombocytopenia. The characteristics of reported patients with immune-mediated thrombocytopenia were as follows:

1. Female,
2. 3rd–8th decades of life,
3. Middle-Eastern and Asian descent,
4. pulmonary tuberculosis most commonly associated (33%),
5. disseminated tuberculosis associated in 19% of cases,
6. tubercular lymphadenitis associated in 19% of cases.

Our case was challenging and unique in several aspects.

1. The patient manifested with classic side effects to several first-line agents used in ATT. Modification of ATT and optimization of therapy were a challenge, and the patient successfully responded to a modified five-drug regimen with INH, PZA, ethionamide, moxifloxacin, and amikacin.
2. The pathology in most of the reported cases was pulmonary tuberculosis. Our patient had tuberculosis-related immune thrombocytopenia in the setting of disseminated tuberculosis (19% association).
3. While most of the cases reported in the world literature were middle-aged females, our patient was a young, adolescent male.

## 4. Conclusion

The incidence of tuberculosis continues to rise due to evolution of bacteria resistant to anti-tuberculous agents and spread of human immunodeficiency virus (HIV) worldwide. Tuberculosis has varied manifestations, and the clinicians must also recognize tuberculosis-mediated immune thrombocytopenia as a manifestation of the disease. The phenomenon has also been recently reported in a child for the first time [15]. The pathophysiologic mechanisms behind it remain unclear. Adherence to anti-tuberculous therapy with initiation of immunomodulatory therapy defines the treatment of this specific condition.

## Figures and Tables

**Table 1:** Haematological manifestations in patients with tuberculosis in various studies.

No.	Haematological changes	Limit	Pulmonary Tuberculosis (in %)	Miliary Tuberculosis (in %)
1	Anemia	<13 g/dl in males; <11 g/dl in females	16–76	52–72
2	Leucopenia	<4000/ml	1.5–4	9–32
3	Leucocytosis	>11000/ml		
4	Lymphocytopenia	<1500/ml	8–31	87–100
5	Neutrophilia	>7000/ml	29–57	20–60
6	Monocytopenia		29–60	4–6
7	Thrombocytopenia	<150000/ml	Rare	23–42
8	Thrombocytosis	>4000000/ml	52	Rare
9	Pancytopenia		Rare	

Reference: [1]
